# The StarT back screening tool and a pain mannequin improve triage in individuals with low back pain at risk of a worse prognosis – a population based cohort study

**DOI:** 10.1186/s12891-019-2836-1

**Published:** 2019-10-22

**Authors:** Emma Haglund, Ann Bremander, Stefan Bergman

**Affiliations:** 1 School of Business, Engineering and Science, Halmstad University, Halmstad, Sweden; 2 Spenshult Research and Development Center, Halmstad, Sweden; 3 Department of Clinical Sciences, Lund, Section of Rheumatology, Lund University, Lund, Sweden; 4 Department of Regional Health Research, University of Southern Denmark, Odense, Denmark; 5 Danish Hospital for Rheumatic Diseases, University Hospital of Southern Denmark, Sønderborg, Denmark; 6 Primary Health Care Unit, Department of Public Health and Community Medicine, Institute of Medicine, The Sahlgrenska Academy, University of Gothenburg, Gothenburg, Sweden

**Keywords:** Low back pain, Chronic widespread pain, Multisite pain, Population-based cohort, Prognostic indicators, Questionnaire

## Abstract

**Background:**

The STarT Back Screening Tool (SBT) identifies patients with low back pain (LBP) at risk of a worse prognosis of persistent disabling back pain, and thereby facilitates triage to appropriate treatment level. However, the SBT does not consider the pain distribution, which is a known predictor of chronic widespread pain (CWP). The aim of this study was to determine if screening by the SBT and screening of multisite chronic widespread pain (MS-CWP) could identity individuals with a worse prognosis. A secondary aim was to analyze self-reported health in individuals with and without LBP, in relation to the combination of these two screening tools.

**Methods:**

One hundred and nineteen individuals (aged 40–71 years, mean (SD) 59 (8) years), 52 with LBP and 67 references, answered two screening tools; the SBT and a pain mannequin – as well as a questionnaire addressing self-reported health. The SBT stratifies into low, medium or high risk of a worse prognosis. The pain mannequin stratifies into either presence or absence of CWP in combination with ≥7 painful areas of pain (0–18), here defined as MS-CWP (high risk of worse prognosis). The two screening tools were studied one-by-one, and as a combined screening. For statistical analyses, independent t-tests and Chi-square tests were used.

**Results:**

Both the SBT and the pain mannequin identified risk of a worse prognosis in individuals with (*p* = 0.007) or without (*p* = 0.001) LBP. We found that the screening tools identified partly different individuals at risk. The SBT identified one individual, while the pain mannequin identified 21 (19%). When combining the two screening methods, 21 individuals (17%) were at high risk of a worse prognosis. When analyzing differences between individuals at high risk (combined SBT and MS-CWP) with those at low risk, individuals at high risk reported worse health (*p* = 0.013 - < 0.001).

**Conclusions:**

Both screening tools identified individuals at risk, but they captured different aspects, and also different number of individuals at high risk of a worse prognosis. Thus, using a combination may improve early detection and facilitate triage to appropriate treatment level with multimodal approach also in those otherwise missed by the SBT.

## Background

Low back pain (LBP) is one of the world’s leading health problems [1–3]. The point prevalence is around 40% in the western world. The symptoms often cause activity limitations and participation restrictions, even though the condition is usually transient [4]. Primary health care is often the first point of care for patients with LBP. Most individuals with LBP have a condition defined as non-specific, in contrast to serious LBP pathology or nerve pathology [5]. Pain in general can be categorized as acute or chronic; it is considered chronic if the pain has been present for at least three months [6]. Besides the individual suffering, LBP can cause an economic burden to the individuals and society [2].

The prevalence of chronic LBP (CLBP) is estimated to affect approximately 23% of the population, but is a more complex condition [7, 8]. It is also known that individuals who once suffered with LBP have recurring episodes of LBP more often over the course of their lives than those who have never had LBP [9]. Knowledge of fundamental prognostic factors will help to understand an individual’s risk of a poorer prognosis and chronic condition. Heavy physical work demands and low physical function are physical risk factors for developing CLBP [10]. Other factors of importance are older age, low general health, stress symptoms, radiating pain, high pain intensity, previous sick-leave due to LBP and low education level [10–12]. The biopsychosocial approach, in which underlying social and psychological factors have also been found to be important, may further increase the knowledge concerning CLBP. Low job satisfaction, low social support at work, sadness and depression are some of these risk factors [13–15]. Kinesiophobia, feelings of fear-avoidance of work activities, physical activities, and catastrophizing (imagining a situation worse than it is) are other factors that seems to influence risk of a worse prognosis and the development of CLBP [15–17]. There is also growing evidence of an association between LBP, concomitant pain in other regions of the body, and the risk of developing more generalized pain [18]. Widespread pain (axial, present in both sides of the body, in upper and lower limb), as well as multisite pain (seven or more anatomical sites), increases the risk of developing a chronic condition [19–21]. An overlap between multisite and chronic widespread pain (CWP) has also been shown [22].

Clinicians need easy-to-use instruments to identify different subgroups of patients with LBP, taking into account early prognostic factors of worse prognosis. The screening instrument STarT (Subgroups for Targeted Treatment) Back Screening Tool (SBT) is a short and easy-to-score tool that identifies individuals at risk of a worse prognosis in order to facilitate triage to appropriate treatment level [23]. The SBT score stratifies individuals to low, medium or high risk of a worse prognosis. Low risk implies management with general advice, medium risk evidence-based physiotherapy and the high-risk group cognitive behavioral therapy. The SBT takes into account known risk factors such as radiating pain, activity limitations, kinesiophobia and catastrophizing, and has shown predictive value for functional improvements two months after a visit to primary care [24]. When using the SBT for triaging to different risks and treatments in primary care settings, in comparison with a control group not using the SBT, the stratified approach has been shown to improve outcome measures such as pain intensity, catastrophizing, fear, anxiety, depression, general health and absenteeism [25]. However, the SBT does not capture all of those at high risk [26, 27]. Thus, the instrument may need supplemental information about the distribution of pain or if the pain is multisite or not.

The American College of Rheumatology’s (ACR) definition of CWP [20] has been used in epidemiological studies to classify individuals with pain symptoms into subgroups [22]. The definition states that CWP is pain (i) for at least three months during the past 12, (ii) distributed in the axial skeleton, (iii) in both sides of the body, and (iv) in the upper and lower limbs. In a modification of the ACR’s diagnostic criteria in pain syndromes such as fibromyalgia, the widespread pain index has been used to assess if patients have multisite pain or not. Seven or more regions of pain were considered as worse severity of fibromyalgia [21]. Pain mannequins have been used in epidemiological studies and are considered valid and reliable measures for the assessment of pain distribution [28, 29]. Screening for physical and psychosocial risk factors together with pain related symptoms by using STB, and by adding the pain mannequin aim to catch important aspects associated to a worse prognosis of persistent disabling back pain [26, 30].

The SBT does not consider pain distribution or if the pain is multisite or not. This study compared screening by the SBT with screening of multisite chronic widespread pain (MS-CWP) based on a pain mannequin, in a population-based group of individuals with and without back pain. The aim was to analyze to what extent the two screening tools could identify individuals with worse prognosis. A secondary aim was to analyze self-reported health in individuals with and without LBP, in relation to the combination of these two screening tools.

## Methods

This study included a sub group of individuals from a well-established cohort (EPIPAIN) in south west Sweden [19, 29]. EPIPAIN is a population-based longitudinal cohort study. At the start in 1995, EPIPAIN included 3928 individuals representative of the adult population, aged 18–74 years. Since then, there have been four follow-ups (1998, 2003, 2007, 2016). The latest survey in May 2016 was sent to 1832 individuals, whereof 65% (1184) responded. This survey serves as the basis for inclusion in the current study. More details of the EPIPAIN project can be read elsewhere [19, 29]. To answer the research questions the included individuals filled in a more comprehensive questionnaire in connection with a clinical visit that took place sometime in between August to November 2016.

### Subjects

A total of 236 individuals aged 40 to 70 years responding to the 2016 EPIPAIN questionnaire were selected to the present study and invited to take part in clinical tests and to answer an additional questionnaire. This included all 176 individuals with a self-report of back pain for three months or more during the last 12 months. Sixty individuals from those reporting no chronic pain (NCP) during the last 12 months were randomly selected and also invited to the study. Of those invited, 126 individuals (83 with chronic LBP and 43 with NCP) agreed to participate.

**Fig. 1:**
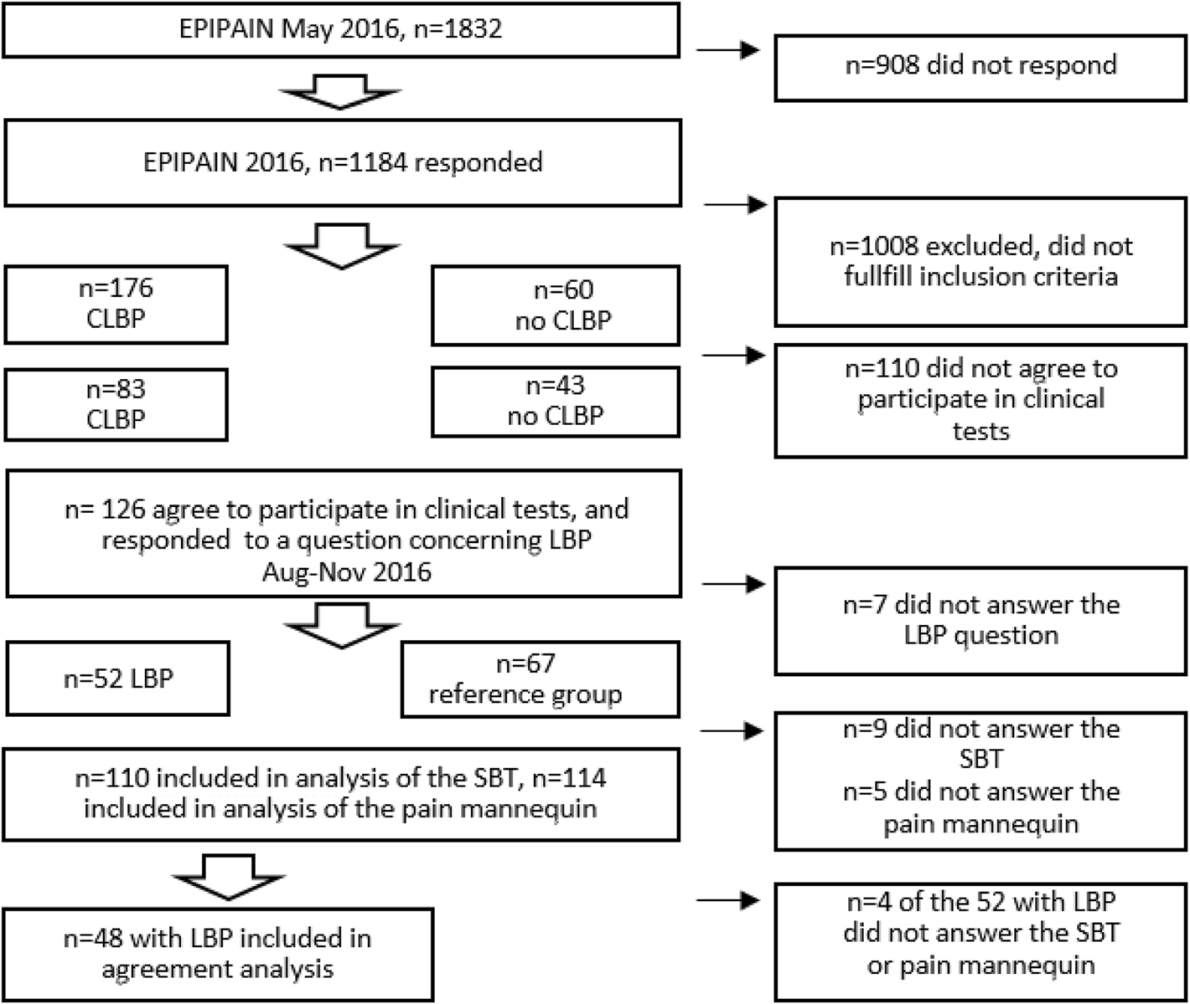
Flow chart showing the participation in the study. EPIPAIN=Population-based longitudinal cohort study. CLBP = chronic low back pain; reports of pain for three months or more during the last 12 months and LBP according to the pain mannequin. STB=STarT Back Screening Tool

To have a current knowledge of the LBP status in connection to the clinical visit, three to six months after the EPIPAIN questionnaire, the individuals answered the question: *Have you had low back pain during the last week? (answer yes/no).* The answer to this question formed the two groups, the LBP and the reference group, that were used for the analyses in the study (Fig. 1). This assignment did not take their earlier report of chronic pain into consideration. The current report of chronic pain based on results from the questionnaire filled out in connection with the clinical visit were used for analyses in the study.

### Instruments

#### STarT back screening tool

The questionnaire survey consisted of two screening tools, and several validated self-report instruments assessing the impact of physical and mental health. The primary screening tool was the SBT, classifying individuals into three different risk levels of worse prognosis (risk for persistent disabling back pain) in order to facilitate triage to appropriate treatment [23, 26]. The SBT includes four questions concerning physical risk factors for worse prognosis: (i) referred leg pain, (ii) neck/shoulder pain, (iii) disability in walking, (iv) difficulties in dressing; and five questions concerning psychosocial factors: (i) fear, (ii) anxiety, (iii) catastrophizing, (iv) feelings of depression and (v) bothersomeness. The sum gives a total score of 0–9 (best to worst) which sorts the individuals into low, medium or high risk of a worse prognosis [23, 26]. The SBT has shown acceptable validity and reliability [31–34]. Low risk of a worse prognosis is defined if the overall tool score is ≤3. The medium risk group included individuals with an overall score of ≥4 in combination with psychosocial subscale score < 4. The high-risk group is those individuals with a psychosocial subscale score ≥ 4.

#### Pain mannequin

The second screening tool was a pain mannequin including questions about the experience and distribution of chronic musculoskeletal pain. The pain mannequin was developed within the EPIPAIN project, constructed in accordance with the ACR definition of CWP [20], and found to have good validity and reliability [35]. It has been presented in more detail in previous studies [19, 29]. In the heading of the pain mannequin an explanation stated that the questions refer to aches and pains in joints and soft tissues. An initial question was used to consider if the pain was chronic or not: *Have you experienced any aches or pain lasting more than three months during the past twelve months?* according to ACR definition [20]. A mannequin with predefined body regions [29] followed the question to categorize individuals into CWP, chronic regional pain (CRP) or NCP. Individuals were categorized to CWP if they had experienced pain symptoms for at least three months during the past 12, and reported widespread pain in the mannequin, that is pain in the axial skeleton, in both sides of the body and in upper and lower part of the body. If they had experienced pain symptoms for the same duration but did not report widespread distribution, they were categorized as CRP. Those with no pain experience for at least three months during the past 12 were categorized as NCP [20]. The number of predefined painful body regions (0–18) were also counted [29]. Presence of seven or more painful regions was designated as multisite pain (MS) [19]. A presence of CWP in combination with ≥7 painful regions were designated as MS-CWP. MS-CWP is known to be associated to a more persistent pain condition (worse prognosis) [19].

#### Other questionnaires

Pain intensity the previous week was recorded using a numeric rating scale (NRS), 0–10 (best to worst) [36]. Low back pain in relation to physical function or disability was assessed by the low back pain specific Roland-Morris Disability Questionnaire (RMDQ), 0–24 (no disability to maximum disability) [37]. Self-reported health state was assessed by the generic EuroQol-5 domain questionnaire (EQ-5D), 0–1 (no health to full health) [38]. Mental health was assessed by the Hospital Anxiety (HAD-A) and Depression (HAD-D) scales, each of these subscales, scoring 0–21 (no distress to maximum distress) [39]. Kinesiophobia was assessed by the Fear-Avoidance Beliefs Questionnaire, evaluating to what extent physical activity (FABQ-PA, subscale one) and work (FABQ-Work, subscale two) affect the pain experience. The score ranged from 0 to 24 in the FABQ-PA and 0 to 42 in the FABQ-Work, (no fear to high scores of fear and avoidance behavior) [40, 41].

### Statistical analyses

Descriptives of the sample are presented as mean and standard deviation (SD) or as frequencies (%). Independent t-tests or Chi-square were used to describe differences in self-reported health and risk screening in the two groups. Independent t-tests were used to analyze differences in self-reported physical and mental health between individuals with different risk levels based on the combination of the SBT and the pain mannequin. The combination implies that all individuals at high risk captured by the SBT or presence of MS-CWP by the pain mannequin identified as at high risk of a worse prognosis. Thus, individuals at low or medium risk assessed by the SBT, or those showing no MS-CWP, were stratified to low risk of a worse prognosis in the combined screening.

## Results

Hundred twenty-six (53%) of the 236 identified individuals accepted the invitation of a clinical visit. When filling in the EPIPAIN follow-up questionnaire in May 2016, 83 participants (61% women) reported chronic low back pain, while 43 participants (56% women) reported NCP. In those who declined an invitation, men were more prevalent.

At the time for the clinical visit, 52 individuals reported LBP and 67 did not and formed the reference group. Seven did not answer the question about LBP in the last week, and thus 119 were available for analysis. The mean (SD) age of the sample (*n* = 119) was 59 (8) years, ranging between 41 and 71 years, and 75 (60%) were women. There were no differences in age and sex between the two groups (Table 1). Individuals with LBP reported worse physical and mental health and a worse SBT score compared with individuals in the reference group. The LBP group also reported a larger number of painful regions and a higher frequency of CWP than the references (Table 1).

**Table 1:** Self-reported health in individuals with low back pain vs reference group (individuals with no LBP)

	Low back pain (n = 52)	Reference group (*n* = 67)	*p*-value
Age (years)	60 (7)	59 (9)	0.233
Sex (women)	65%	57%	0.337
SBT (0–9)	2.4 (1.5)	0.7 (0.9)	< 0.001
RMDQ (0–24)	6.6 (4.7)	2.0 (2.1)	< 0.001
EQ5D (0–1)	0.69 (0.21)	0.87 (0.14)	< 0.001
FABQ PA (0–24)	8.4 (5.9)	5.5 (5.4)	< 0.001
FABQ work (0–42)	15.0 (12.3)	8.5 (8.2)	< 0.001
HAD anxiety (0–21)	7.7 (2.9)	5.1 (3.4)	< 0.001
HAD depression (0–21)	3.7 (1.8)	2.2 (2.0)	< 0.001
Pain NRS (0–10)	5.1 (2.1)	1.2 (2.4)	< 0.001
Regions with pain (0–18)	5.0 (4.2)	1.9 (3.0)	< 0.001
CWP at inclusion	53%	18%	< 0.001

Presented as mean (SD) or frequencies and *p*-values. SBT = STarT Back Screening Tool (best-worst), RMDQ = Roland-Morris Disability Questionnaire (best-worst), EQ5D = EuroQol 5-domain (worst-best), FABQ = Fear-Avoidance Beliefs Questionnaire for physical activity and work (best-worst), HAD = Hospital Anxiety and Depression scale (best-worst), NRS=Numeric Rating Scale, LBP = Low Back Pain, CWP = Chronic widespread pain, CLBP=Chronic LBP

### Risk assessment by the different screening tools

When stratifying individuals into low, medium or high risk of a worse prognosis by the SBT, 41 individuals (80%) in the LBP group and 58 in the reference group (98%) were stratified into the low risk group. Nine individuals (18%) with LBP were stratified as medium risk and one as at high risk of a worse prognosis. There was a statistically significant difference in risk distribution based on the SBT between the LBP group and references, *p* = 0.007 (Table 2). The pain mannequin showed a statistically significant difference in risk distribution between the LBP and the reference group, *p* < 0.001. In the LBP group, 16 individuals (33%) reported MS-CWP and were considered as at high risk of a worse prognosis. Four of the references (6%) also reported MS-CWP, but did not report LBP last week (Table 2). Nine individuals did not complete the SBT and five did not complete the pain mannequin, thus these analyses were based on 110 and 114 individuals respectively.

**Table 2:** Screening by the STarT Back Screening Tool (SBT) (*n* = 110) and by pain mannequin (*n* = 114)

	Low back pain	Reference group
SBT – Low risk	41 (80)	58 (98)
SBT – Medium risk	9 (18)	1 (2)
SBT – High risk	1 (2)	0
No MS-CWP	33 (67)	61 (94)
MS-CWP	16 (33)	4 (6)

Screening by the SBT for low, medium or high risk of a worse prognosis and by pain mannequin for multisite chronic widespread pain (MS-CWP) or not (No MS-CWP) in both groups (LBP vs. reference group). Presented as number of individuals and percent (%). MS-CWP = multisite pain in ≥7 sites and chronic widespread pain distribution

Forty-eight individuals with LBP were available for analysis of the agreement between the two screening methods (SBT vs. pain mannequin). Only one individual was at high risk as captured by the SBT. Using the pain mannequin, 16 (33%) had MS-CWP and were defined as at high risk of a worse prognosis. Eleven individuals (23%) reported MS-CWP in the SBT low risk-group and four individuals reported MS-CWP in the medium risk-group (Table 3).

**Table 3:** Observed agreement between the STarT Back Screening Tool and the pain mannequin, *n* = 48

	STarT Back Screening Tool		
	Low risk (*n* = 39)	Medium risk (*n* = 8)	High risk (n = 1)
No MS-CWP (*n* = 32)*	28	4	0
MS-CWP (*n* = 16)**	11	4	1

Analysis based on individuals with low back pain. Presented as number of individuals

* = No multisite chronic widespread pain (CWP). ** = Multisite-CWP

### Differences in self-reported health

Sixteen individuals (31%) were included in the high-risk group for a worse prognosis when the two screening tools were combined. Individuals at high risk reported worse health with statistically significant differences in reports of anxiety (HAD-A), depression (HAD-D), and pain intensity (NRS) when compared with those at low risk. The trend was also uniform, but not statistically significant, for their reports of physical function (RMDQ) and self-reported health (EQ5D) (Table 4).

**Table 4:** Differences in self-reported physical or mental health, *n* = 52

	Lower risk in combined screening (*n* = 36) Mean (SD)	High risk in combined screening (n = 16) Mean (SD)	*p*-value
RMDQ (0–24)	5.8 (4.1)	7.8 (5.3)	0.228
EQ5D (0–1)	0.72 (0.18)	0.63 (0.27)	0.155
FABQ PA (0–24)	8.6 (5.5)	7.9 (6.8)	0.701
FABQ work (0–42)	12.6 (11.6)	20.1 (14.4)	0.066
HAD anxiety (0–21)	7.2 (3.1)	9.1 (1.7)	0.026
HAD depression (0–21)	3.3 (1.9)	4.6 (1.3)	0.016
Pain NRS (1–10)	4.5 (2.0)	6.3 (1.9)	< 0.001

Analysis based on the triage by the combination of the SBT and pain mannequin in individuals with low back pain. SBT = STarT Back Screening Tool. Lower risk in combined screening = SBT low and medium risk or no Multisite Chronic Widespread Pain (MS-CWP). High risk in combined screening = SBT high risk or MS-CWP. RMDQ = Roland-Morris Disability Questionnaire (best-worst), EQ5D = EuroQol 5-domain (worst-best), FABQ = Fear-Avoidance Beliefs Questionnaire for physical activity and work (best-worst), HAD = Hospital Anxiety and Depression scale (best-worst), NRS = numeric rating scale (best-worst)

## Discussion

Back pain is a major health problem for society as well as one of the leading causes of disability (3). Thus, it is important in clinical practice to identify individuals at risk of worse prognosis early, and to develop a triage system for interventions identifying those in need of cognitive behavioral therapy. In this sub sample of a population-based cohort of individuals with and without self-reports of LBP, the risk of a worse prognosis was compared between the two different screening tools. Both the SBT and the pain mannequin discriminated between different risk levels of worse prognosis in individuals with or without LBP. A combination of the two screening tools seemed to also capture more individuals at risk of worse prognosis than if the SBT were used alone. Thus, the combination of the two screening tools may improve the ability to early detect those at risk of a worse prognosis and facilitate triage to appropriate treatment level in a clinical setting.

### Risk assessment by the different screening tools

We found significant differences in risk distribution between individuals with and without LBP for both the SBT and the pain mannequin. However, the SBT did not capture many at high risk (< 1%). In the original study where the SBT was developed, 15% were allocated to the high-risk group [23]. Other studies have found frequencies in the range of 0–32% at high risk of a worse prognosis when using the SBT [24, 25, 34, 42–44]. In an earlier study, no-one at high risk was captured by the SBT, despite inclusion of health-care seeking patients [27]. We also found a higher frequency of individuals at low risk identified by the SBT (about 90%) in comparison with earlier research where the reported frequencies of low risk individuals range from 26 to 66% [23–25, 27, 34, 42–44]. One reason for these dissimilarities could be explained by the different inclusion criteria and samples used in the different studies.

The SBT was primary developed, validated and proven as acceptable for use for individuals seeking primary health-care for their LBP [23, 34, 43], while this study used a population-based sample and the individuals were included regardless of the need for a health-care visit. To our knowledge, no other study has tested the SBT in a comparable setting of individuals from the general population. Therefore, it is not remarkable that our sample reported better health than described in earlier studies. For example, the mean RMDQ score for the total sample was found to be less than 5 in the current study, while others have reported a mean of 9 or higher [23–25, 43]. Also, the mean of pain intensity (score > 5) [25, 27, 34], and the SBT score (score 3–4) [15, 23, 27, 34, 42] were higher in earlier studies (compared with our 3.9 for pain and 1.8 for the SBT). Based on the SBT, most of the individuals were screened as at low risk, both individuals with LBP (80%) and references (98%). When combining the screening tools and studying self-reported health, the RMDQ almost reached a mean of 8 in those with high risk of a worse prognosis, which is more in line with earlier data. In the LBP group, 8% scored zero (lowest) in the SBT, while 53% of the reference group scored zero. None of the individuals scored 9 (the highest possible score) in the SBT in either of the groups. These results are in line with a recent study, where the comparable figures are 6% scoring 0, and no-one scoring 9 [27].

In the current study, the SBT only identified one individual at high risk of worse prognosis, also captured when screening by the pain mannequin. Nearly one-third of those classified at low risk by the SBT and half of those classified as medium risk turned out to have had a simultaneous multisite chronic widespread pain (MS-CWP) by the pain mannequin. Multisite widespread pain has been found to influence the probability of worse prognosis and/or CLBP [30]. Even so, pain intensity or the distribution of pain are not considered in the nine questions of the SBT [23]. The risk of missing individuals with low SBT scores but high risk according to pain distribution, led to our hypothesis that a combined use of these screening tools would identify individuals at high risk of a worse prognosis more accurately. Conversely, half of those screened as at low risk by the pain mannequin were identified as medium risk by the SBT. This clearly illustrates how the two instruments capture different aspects, and why a combination of the two might capture a more appropriate sample in need of cognitive behavioral therapy and multidisciplinary interventions.

### Differences in self-reported health

We found a clear difference in self-reported health between individuals with LBP and those in the reference group, even if the result indicates that symptoms of pain are fluctuating slightly over time. The current study also found that, when combining the screening tools in order to allocate individuals into a low or high-risk of a worse prognosis, the low-risk group had significantly better self-reported physical and mental health concerning anxiety, depression, and pain intensity. There was also a trend towards differences in their reports of physical function, health-related quality of life and fear-avoidance. These important differences did not reach statistical significance, however, possibly due to a small sample size. In line with this, earlier research has described worse health in individuals with LBP than in the general population [45, 46] and those at low risk of a worse prognosis reported better health [24, 25, 27, 42, 44]. Other studies have shown that individuals with high levels of pain also had worse reported health [10, 47]. Thus, both screening tools used in the combined screening seemed to be able to identify different factors of worse prognosis. However, to use both instruments independently may lead to far too many being classified as at high risk of a worse prognosis. Thus, using a combination may improve the ability to capture also those with multisite widespread pain missed by the SBT.

### Strengths and limitations

The observed agreement between the two screening tools and their ability to stratify individuals into different levels of risk of a worse prognosis (Table 3) clearly illustrates how the tools to some extent identify different individuals. Compared with using the SBT solely, the pain mannequin added individuals who otherwise would have been missed. An early identification of those at risk of a worse prognosis is of great value for both individuals and society, regardless of the underlying prognostic factor.

A limitation is that the analyses were based on a sample from the general population. It makes it somewhat difficult to compare the results with other studies that used the SBT in a clinical setting. Most studies testing the SBT used samples from health-seeking individuals in primary care, as the SBT was developed for that setting. However, not all individuals with LBP seek health care [8], and the heterogeneity of the current sample could well represent the general population and credible generalizations from this study can therefore be made. Another limitation is the low number of individuals with a high risk of a worse prognosis based on the SBT. To better understand the benefits, the combination of these two screening tools needs to be tested in clinical practice in primary health care in a randomized controlled study. In future studies it is also important to consider if it is better to upgrade individuals identified at high risk by the pain mannequin if they have a medium risk of a worse prognosis by the SBT, otherwise a too large number of individuals may be triaged to treatment with cognitive behavioral therapy.

## Conclusion

The SBT and the pain mannequin were complementary in identifying partly different individuals with LBP that could have a higher risk of a worse prognosis and are in need of a more comprehensive treatment. A combination of the two instruments may facilitate a triage to the appropriate treatment level, as individuals who are at high risk due to multisite chronic widespread pain are missed by the SBT, but picked up by the pain mannequin. This combined screening method needs to be tested in a clinical setting.

## Data Availability

The data from the current study are not publicly available according to the ethical approval and patients’ informed consent, but could be available from the corresponding authors upon reasonable request.

## Abbreviations

CRP: Chronic regional pain
CWP: Chronic widespread pain
EPIPAIN: The epidemiology of pain cohort
EQ-5D: EuroQol-5 domain
FABQ: Fear-Avoidance Beliefs Questionnaire
HAD Scale: Hospital Anxiety (HAD-A) and Depression scale
LBP: Low back pain
MS-CWP: Multisite chronic widespread pain
NCP: No chronic pain
NRS: Numeric Rating Scale
RMDQ: The Roland-Morris Disability Questionnaire
SBT: Subgroups for Targeted Treatment Back Screening Tool
SD: Standard deviation
